# Development of Dual Inhibitors against Alzheimer's Disease Using Fragment-Based QSAR and Molecular Docking

**DOI:** 10.1155/2014/979606

**Published:** 2014-06-12

**Authors:** Manisha Goyal, Jaspreet Kaur Dhanjal, Sukriti Goyal, Chetna Tyagi, Rabia Hamid, Abhinav Grover

**Affiliations:** ^1^Apaji Institute of Mathematics & Applied Computer Technology, Banasthali University, Tonk, Rajasthan 304022, India; ^2^School of Biotechnology, Jawaharlal Nehru University, New Delhi 110067, India; ^3^Department of Biochemistry, University of Kashmir, Srinagar 190006, India

## Abstract

Alzheimer's (AD) is the leading cause of dementia among elderly people. Considering the complex heterogeneous etiology of AD, there is an urgent need to develop multitargeted drugs for its suppression. *β*-amyloid cleavage enzyme (BACE-1) and acetylcholinesterase (AChE), being important for AD progression, have been considered as promising drug targets. In this study, a robust and highly predictive group-based QSAR (GQSAR) model has been developed based on the descriptors calculated for the fragments of 20 1,4-dihydropyridine (DHP) derivatives. A large combinatorial library of DHP analogues was created, the activity of each compound was predicted, and the top compounds were analyzed using refined molecular docking. A detailed interaction analysis was carried out for the top two compounds (EDC and FDC) which showed significant binding affinity for BACE-1 and AChE. This study paves way for consideration of these lead molecules as prospective drugs for the effective dual inhibition of BACE-1 and AChE. The GQSAR model provides site-specific clues about the molecules where certain modifications can result in increased biological activity. This information could be of high value for design and development of multifunctional drugs for combating AD.

## 1. Introduction

Alzheimer's disease (AD) is an irreversible chronic brain disorder among elderly people [1–3]. AD is characterized by steady cognitive impairment, memory loss, and decline in language. It is one of the leading causes of death in the world. For instance, it was estimated that 5.2 million Americans of all ages were suffering from AD in 2013 making it the sixth leading cause of death in the United States (Alzheimer's association; http://www.alz.org/). The devastating pathological hallmarks of AD are extracellular accumulation of neurotoxic amyloid *β* (A*β*) peptides [4], loss of the presynaptic markers of the cholinergic system in the brain, mitochondrial dysfunction, and formation of dense neurofibrillary tangles of hyperphosphorylated tau protein in the central nervous system [5–7].

Most of the U.S. Food and Drug Administration approved drugs are available for the symptomatic treatment of AD. Among these drugs, donepezil, tacrine, rivastigmine, and galantamine are based on cholinergic hypothesis [8–11]. Furthermore, memantine is an antagonist drug of N-methyl-D-aspartate receptor [12–14]. However, the observable toxic issues such as hepatotoxicity, vomiting, diarrhea, and nausea forced these drugs to move out from the pharmaceutical market [15]. Moreover, medicational pharmacokinetic effects of these drugs are just for marginally alleviating the symptoms and not to have interruption in neurodegenerative cascade which is the root pathophysiology of AD [16–18]. Considering the complex heterogeneous etiology of AD, modulation of one enzyme might not be sufficient enough for the effective treatment of AD. Therefore, the present day research in AD drug development is shifting towards identification and design of multitargeted novel molecules instead of single targeted molecules for the long term suppression of AD. For instance, Piazzi et al. report AChE inhibitor purposely designed to bind at both the catalytic and the peripheral sites of the human enzyme [19].

Most of the experimental evidences suggest that deposition of amyloid plaques in the brain of Alzheimer's patients is the key factor of pathogenic cascade of the disease [16, 20]. A*β*, which is the core component of the amyloid plaques [15], is produced by subsequent cleavage of a large transmembrane protein—amyloid precursor protein (APP)—by two different proteolytic enzymes *β*- and *γ*-secretase [21]. The complete biochemical mechanism of proteolytic cleavage depends on the protein-protein interactions between APP and *β*-amyloid cleavage enzymes (BACE-1) [22]. Blocking the interface between these protein interactions has huge therapeutic potential for slowing down the long term progression of AD. It has been reported that acetylcholine esterase (AChE) also plays an important role in accumulation of A*β* and acts as a promoter of A*β* fibril production [23]. This activity of AChE is associated with its peripheral anionic site (PAS). Since BACE-1 plays a major role in the initiation of neuropathological cascade of plaque formation and AChE accelerates A*β* deposition in brain, both of these enzymes hold considerable promise as therapeutic targets of AD. Thus, dual target directed strategy is more likely to show comprehensive obliteration of AD in synergistic manner. Multitarget drugs are more efficient as they prevent unwanted compensatory mechanisms, which might result in cellular redundancy, from developing [24].

Discovery of small molecules for targeting protein-protein interfaces beholds enormous challenges and is accounted by various factors, namely, shape of typical protein-protein interface and flexibility of proteins among others. To speed up the drug discovery process, various fast and accurate computational methods have been illustrated which assist the development of novel therapeutic drugs to interrupt the interaction between proteins [25, 26]. Usage of quantitative structure activity relationship- (QSAR) based approaches is worthwhile when knowledge of ligand molecules for a particular target is available. Group-based QSAR (GQSAR) is one of the most recent and effective ligand-based drug designing approaches which uses descriptors evaluated specifically for the substituent groups or fragments of the ligands. This approach identifies the specific sites where the groups need to be modified for designing optimized molecules with enhanced biological activity [27]. GQSAR model can be developed by applying statistical methods like partial least square (PLS), principle component regression, multiple regression, continuum regression, and k-Nearest Neighbour on a series of congeneric compounds in order to gain insights into the effects of descriptors on their biological activity [27, 28].

Herein, our attempts are focused on the discovery of novel small molecules that can compete to bind with one of the interacting proteins with higher binding affinity in order to disrupt the interactions between APP and BACE-1 and simultaneously are able to bind to the PAS site of AChE. Present study describes a detailed GQSAR analysis on 1,4-dihydropyridine (DHP) derivatives, reported as potential inhibitors of BACE-1 [4], in order to elucidate the structural features of the molecular fragments of these molecules that lay significant contribution towards their biological activity. GQSAR model was further used to develop a combinatorial library of novel molecules followed by their activity prediction. Mechanistic analysis of binding modes of these identified leads within the active site of both targets was performed using docking studies. Thus, our study delineates identification of novel leads having dual inhibiting effects due to binding to both, BACE-1 and the PAS of AChE.

## 2. Materials and Methods

### 2.1. Biological Dataset

A biological data set of 20 compounds of DHP derivatives was chosen in the present study to carry out the GQSAR analysis. DHP were found to have strong inhibitory capability against BACE-1 [4]. The experimentally reported inhibitory activity [IC_50_ (*μ*M)] of all the 20 compounds was converted into pIC_50_ [−log_10_ IC_50_], which was then subsequently used as response or dependent variable for GQSAR model building. The 2D structures of compounds were drawn using Marvin Sketch (v 5.12.1, ChemAxon) [21]. 2D chemical structures of DHP analogues along with their biological activities are presented in Table 1. Molecules were converted into 3D format and then energetically optimized using Vlife Engine module of Vlife Molecular Design Suite (Vlife MDS) [29]. The optimized molecules were generated using Merck Molecular force field, distance dependent function, and energy gradient of 0.01 kcal/mol.

### 2.2. Fragmentation and Descriptor Calculation

All molecules considered here had a common DHP scaffold and 4 substitution sites where different R-groups were attached. On the basis of different R-groups, each molecule was divided into 4 fragments or groups in order to perform GQSAR analysis. Optimized dataset of all molecules was considered for GQSAR analysis on the basis of common DHP template. A total of 705 physicochemical descriptors were calculated for various groups present at each substitution site using Vlife MDS. These included 2D descriptors such as element count, extended topological indices, Merck molecular force field atom type count, and electrotopological and alignment independent descriptors among others [30]. Independent variable calculation was further followed by removal of invariable columns containing constant values for more than 90% molecules, which finally resulted in 311 independent variables from the large pool of descriptors.

### 2.3. Selection of Test Set and Training Set

With an aim to develop a GQSAR model, the dataset was split into two optimal training and test sets using random selection method. The robustness of these sets was evaluated by generating unicolumn statistical parameters such as mean, standard deviation, maximum, and minimum for both test and training sets. The dataset division satisfied the criteria of an appropriate model; namely, the maximum of the test set was less than the maximum of the training set and the minimum of the training set was greater than the minimum of the test set. This analysis validated the selected training and test sets.

### 2.4. GQSAR Model Generation

To select the optimal subset of variables (descriptors) that can significantly correlate with biological activity of molecules from the pool of descriptors, various variable selection methods such as step-wise search algorithm, genetic algorithm, and simulated annealing among others can be used. A number of statistical methods such as partial least square (PLS), multiple regression, and principle component regression can be used for model building. Herein, simulated annealing combined with PLS regression was used to generate the GQSAR model. Simulation of a physical process is known as simulated annealing, which involves heating the system to a high temperature and then gradually cooling it down to room temperature [31]. All the values of statistical parameters for simulated annealing were kept as default. The number of terms (number of descriptors) to be included in the final GQSAR model was kept as 3.

### 2.5. Model Evaluation and Validation

The developed GQSAR model was evaluated using two types of validation—internal and external validations. Internal (cross) validation was carried out using leave-one-out method [32]. Cross-validation coefficient* q* ^2^ was calculated as

(1) $$\begin{array}{c} q^{2}=1-\frac{\sum {\left(y_{i}-{\hat{y}}_{i}\right)}^{2}}{\sum {\left(y_{i}-y_{\text{mean}}\right)}^{2}}, \end{array}$$

where *y*_*i*_ and ${\hat{y}}_{i}$ are the actual and the predicted activity of the *i*th molecule in the training set, respectively, and *y*_mean_ is the average activity of all molecules in the training set.

For external validation of the model, the pIC_50_ values of the test set molecules were predicted and the pred_*r* ^2^ value that provides the statistical correlation between predicted and actual activities of the test set compounds was calculated as follows:

(2) $$\begin{array}{c} \text{pred\_}r^{2}=1-\frac{\sum {\left(y_{i}-{\hat{y}}_{i}\right)}^{2}}{\sum {\left(y_{i}-y_{\text{mean}}\right)}^{2}}, \end{array}$$

where *y*_*i*_ and ${\hat{y}}_{i}$ are the actual and the predicted activity of the *i*th molecule in the test set, respectively, and *y*_mean_ is the average activity of all molecules in the training set.

All these statistical parameters were used to evaluate the quality of the model. Correlation coefficient (*r* ^2^) described the fitness of training set data whereas predictive correlation coefficient (pred-*r* ^2^) was used to evaluate the fitness of test set. Cross-validation coefficient (*q* ^2^) and* F*-test (Fischer's value) showed the statistical significance of the regression model and the standard errors (pred_*r* ^2^_se,* q* ^2^_se, and* r* ^2^_se) gave an idea of the quality and fitness of the model. Low standard error values indicated that the model is absolute and robust. The model is said to be robust and predictive if these statistical parameters satisfy the following conditions:* r* ^2^ > 0.6, pred_*r* ^2^ > 0.5, and* q* ^2^ > 0.6 [33, 34].

### 2.6. Combinatorial Library Generation and Activity Prediction

A combinatorial library was generated using Leadgrow module of Vlife MDS. For library generation a number of substitutions were made using various atoms and groups like alkyl, alkene, acids, aromatic rings, rings, carbonyl, cyanate, –O–CH_3_, –O–C_2_H_5_, amide, benz, and hydrzo at all substitution sites (R1, R2, R3, and R4) of DHP template. The final GQSAR model generated was used for biological activity prediction of the compounds of the combinatorial library.

### 2.7. Docking Studies

The 3D structure of human BACE-1 (resolution: 1.70 Å) was obtained from PDB (PDB ID: 2B8L) [35]. The water molecules and all other heteroatoms were removed from the protein crystal structure. The protein was further prepared using Schrodinger's protein preparation wizard [36]. Conversion of all combinatorial structures to 3D form and further optimization were carried out using LigPrep module of the Schrodinger suite. All possible conformers for each molecule were generated using LigPrep. Docking studies were performed using Glide module of Schrodinger suite by creating a cubic grid (10 × 10 × 10 Å) around the active site residues of BACE-1 that are involved in cleavage of APP. The molecules of combinatorial library with high predicted activity were subjected to high throughput virtual screening (HTVS) protocol followed by Glide's extra precision (XP) docking protocol for futher docking refinement.

### 2.8. Dual Inhibition Effect Studies

Keeping in mind our aim to discover potent novel dual inhibitors of AChE and BACE-1, the above screened molecules were again subjected to docking at PAS site of AChE. This PAS site is involved in accumulation of A*β* in the human brain. Crystal structure of human AChE (resolution: 2.0 Å) was obtained from PDB (PDB ID: 4M0E) [35]. Protein preparation and optimization was done using Schrodinger suite. Selected molecules having high XP scores were then checked for their drug-like properties using Lipinski filters. The two top scoring compounds showing dual inhibitory property were analyzed to observe the molecular mode of interaction between the target proteins and the ligands using ligplot program [37].

## 3. Results and Discussion

Here we have attempted to identify a novel GQSAR model depicting robust statistical correlation between structure and activity of DHP analogues which have been reported as potent suppressors of BACE-1. The adopted strategy initially identified a pool of 311 molecular descriptors to be used as independent variables. The pIC_50_ value was used as the dependent variable. The dataset of 20 compounds was divided into two groups: test set including 5 molecules and training set including the rest of the molecules. The training set was used for model building (Supplementary Table 1 available online at http://dx.doi.org/10.1155/2014/979606).

### 3.1. Dataset Evaluation

Before proceeding towards the next step, evaluation of the chosen test set is always a beneficial option to obtain a good predictive model. This was done by interpreting the unicolumn statistics mentioned in Table 1. Unicolumn statistics are stated in terms of min., max., average, std. dev. (standard deviation), and sum. The min. of test set should be equal or higher than the min. of training set and the max. of test set should be equal or lower than the max. of training set. Here, the dataset was found satisfying the required conditions, thus suggesting that the test set was interpolative. Along with these parameters, average and std. dev. determines the density distribution of both the test and the training sets. Interestingly, in this dataset, higher values of mean and std. dev. for training set indicated the presence of comparably high number of active molecules rather than the inactive ones and the presence of highly distributed activity of the molecules in the training set.

### 3.2. Generated GQSAR Model

**Figure 1:**
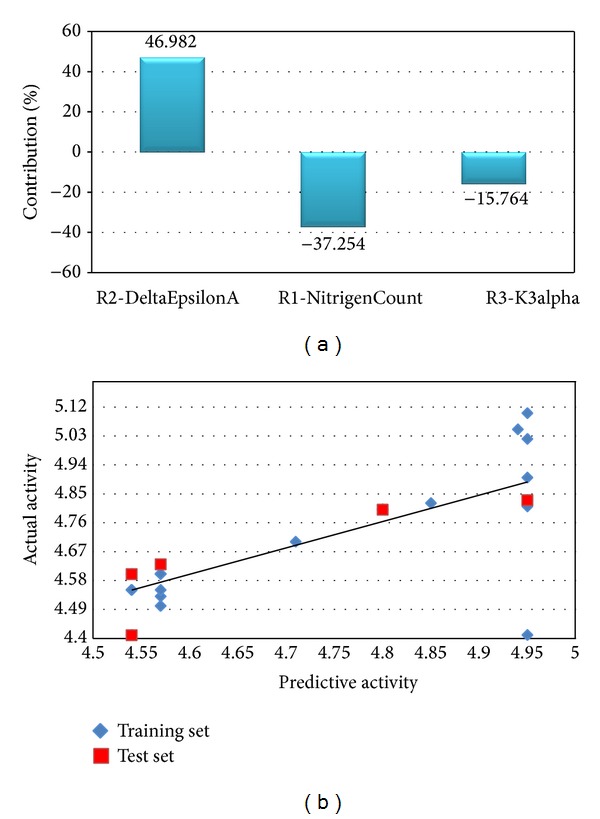
(a) The contribution of descriptors to the enhancement of biological activity of molecules. (b) Linear scatter plot depicting the distance of training and test data points from the regression line.

The GQSAR model was generated using simulated annealing variable selection method in combination with PLS regression model building method. The statistical measurements of generated PLS regression model of GQSAR are summarized in Table 2. PLSR method predicts the correlation between the molecular fields and the inhibitory activity of the compounds [38]. It specifies the linear relationship between dependent variables (pIC_50_) and the predictor variables (descriptors). Predicted activity of the dataset and the values of calculated descriptors for each molecule are mentioned in Supplementary Table 2. The reported GQSAR model can be stated in the form of a polynomial equation as follows:

(3) pIC50=3.48219(R2-DeltaEpsilonA) −0.409885(R1-NitrogensCount) −0.279723(R3-k3alpha)+4.56912,

where R1, R2, and R3 are the 2D descriptors along with their respective coefficient and the last numerical term in this equation is the regression constant. This equation explains that the descriptor DeltaEpsilonA shows positive contribution at substitution site R2 of DHP common moiety. However, the other two descriptors, NitrogensCount and K3alpha at R1 and R3 substitution sites, respectively, contribute negatively towards the biological activity of molecules. The contribution of these descriptors is illustrated in Figure 1. Below is the brief description of these molecular descriptors.

*R2-DeltaEpsilonA*. DeltaEpsilonA falls into the category of extended topochemical atom (ETA) indices which is an extension of topochemically arrived unique parameters [39, 40]. Among the various basic parameters of ETA, DeltaEpsilonA is a measure of contribution of unsaturation and electronegative atom count [41] which is extensively applied for modelling various toxicity end-points in the quantitative domain of structure-activity relationships [42]. Here, it was observed that DeltaEpsilonA showed 46.98% contribution in activity enhancement of molecule when present at R2 site. Originally, R2 site was occupied by three different groups, namely, methylbenzylamine [NH-(*α*) methylBn], benzyl ester (OBn), and acetyl group. 

*R1-NitrogensCount*. This physicochemical descriptor lies in the section of element count descriptors. As the name suggests, it indicates the number of nitrogen atoms present in a compound. This descriptor was observed to provide a 37.25% negative contribution at R1 substitution site which was originally engaged with different alkyl groups. 

*R3-k3alpha*. The Kier and Hall Kappa molecular shape indices are intended to capture the overall aspects of molecular shape [43]. Third order Kappa Alpha (K3alpha) shape index is a subset of Kappa indices and the information encoded in it specifically refers to attributes of the shape of molecule. In present GQSAR model, K3alpha was found to have 15.74% negative participation at R3 substitution site for the enhancement of biological activity of molecules. This site was originally occupied by sulphonamide group, amide group, and ester group.

### 3.3. GQSAR Model Validation

**Figure 2:**
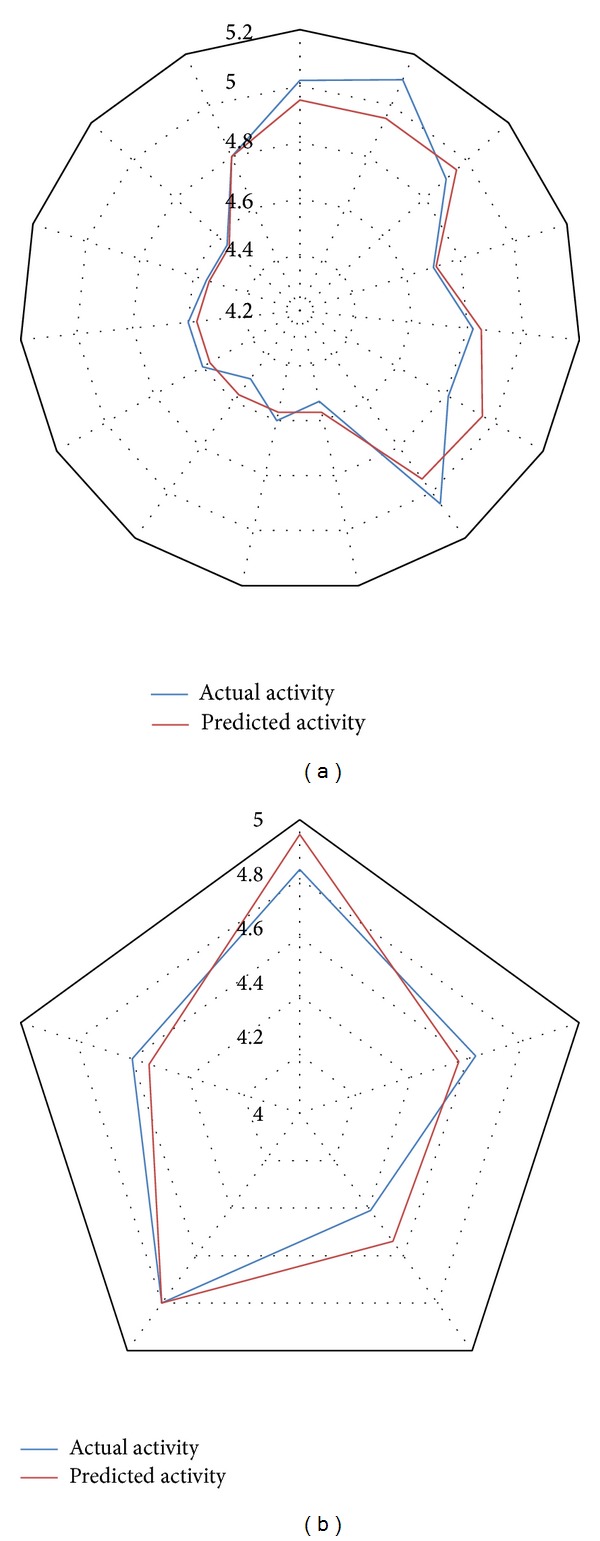
(a) Radar plot showing fitness of predicted and actual activity values of training set. (b) Radar plot exploring fitness of predicted and actual activity values of test set.

The quality of the GQSAR model was judged on the basis of standard values of statistical parameters calculated during model generation. In this study, the convincing parametric values for GQSAR model were observed in terms of correlation coefficient* r* ^2^ (0.8514), predicted correlation coefficient pred_*r* ^2^ (0.7525), cross-correlation coefficient* q* ^2^ (0.6817), low standard error* r* ^2^_se (0.0847),* q* ^2^_se (0.1239), and pred_*r* ^2^se (0.0976) which implied that the model can be considered stable and accurate. Moreover, high values of other statistical parameters like* F*-test (34.3899) provided additional support that the model was significant and robust with minimum chance of failure. For better understanding of the relationship between the structural features of DHP derived molecules and their biological activity, two different graphical representations of predicted and actual activity values are shown in Figures 1(b) and 2. Two separate radar plots describe the fitness of predicted over actual values for training and test sets, respectively, and the linear scatter plot depicts the distance of training and test data points from the regression line which relatively gives an idea about the difference between actual and predicted activity values of both sets.

### 3.4. Combinatorial Library Preparation and Activity Prediction

**Figure 3:**
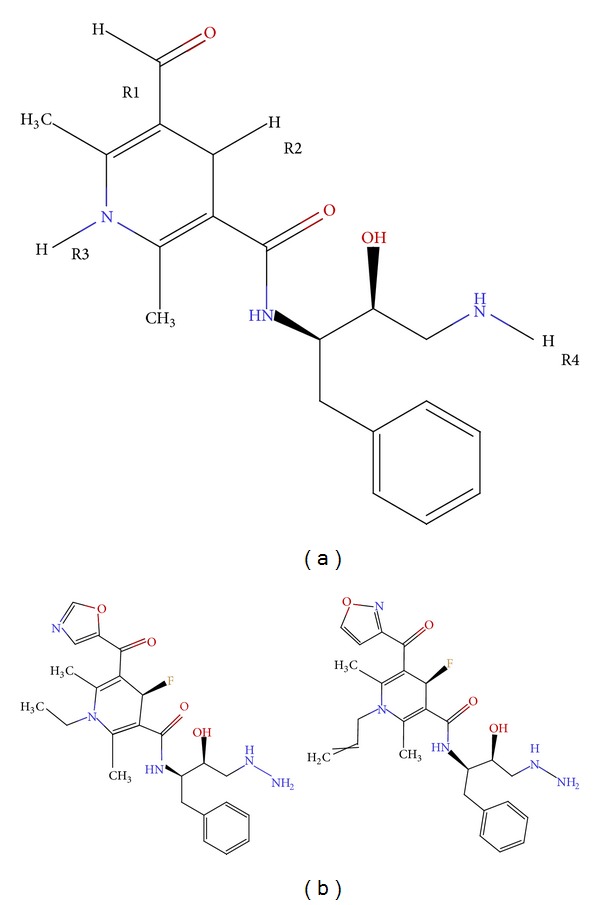
(a) 2D structure of common moiety of DHP derivatives. (b) 2D structures of selected molecules (EDC and FDC) possessing dual inhibitory property.

The common moiety (Figure 3(a)) of DHP derivatives was taken into account for generation of the combinatorial library of novel compounds. This works by putting different chemical groups or atoms at four different substitution sites, namely, R1, R2, R3, and R4 of common template. At R1 site, different groups like alkyl, vinyl, and allyl acetate were added. At R2 site, alkyl, phenyl, pyrrole, benzopyrrole, thiophenone, oxazolyl, pyrimidinyl groups, and aromatic rings were placed. Number of different atoms as in S, N, H, He, Li, F, alkyl groups, and other groups such as –O–CH_3_, –O–C_2_H_5_, amide, cyanide, cyanate, isocyanate, –C=N, –N=C, azo, and hydrazo were added at R3 site. R4 site was filled with atoms (O, N, F, Be.) and different cyclic rings. All possible combinations of different chemical groups at four substitution sites resulted in a large combinatorial library of 86,400 compounds. The complete library was than subjected to biological activity prediction using the generated GQSAR model. 3405 compounds possessing higher activity values (>5.0) were chosen for further binding analysis against AChE and BACE-1. Compound 4 was observed to have maximum activity (6.51) in which R1 site was occupied by 2-thiophene group; R2 site was found to have F, with ethyl group and N at R3 and R4 site, respectively. Surprisingly, approximately all the high activity molecules were found to bear F atom at R2 site suggesting that the presence of F atom at R2 site plays a crucial role in activity enhancement. Therefore, constant value of 0.557 for extended topochemical descriptor R2-DeltaEpsilonA was observed. The constant low values of 1 and 0 for negatively contributing descriptors R1-NitrogensCount and R3-K3alpha depicted their role in activity enhancement.

### 3.5. Docking Analysis

Docking studies for 3405 molecules of combinatorial library were carried out against AChE and BACE-1. To filter out the chemically correct structures, molecules were converted into 3D format and then optimized using LigPrep module of Schrodinger suite which reduced the number of molecules for further analysis to 3238. Among these molecules, a total of 1310 and 1482 compounds having good binding affinity for BACE-1 and AChE, respectively, were identified using HTVS. After HTVS, the highest docking scores for both targets, BACE-1 and AChE, were found to be −10 kcal/mol and −12 kcal/mol, respectively. Compounds with Glide score above −8 kcal/mol for BACE-1 and −6 kcal/mol for AChE were then subjected to XP protocol for further refinement of Glide score. The two top scoring compounds showing dual inhibitory property against both targets were selected for further evaluation of their mechanistic molecular mode of interaction with the target proteins.

### 3.6. Interaction Mode Analysis of Docked Complexes

The two top scoring compounds, namely, (4R)-1-ethyl-4-fluoro-N-[(2R,3S)-4-hydrazinyl-3-hydroxy-1-phenylbutan-2-yl]-2,6-dimethyl-5-(1,3-oxazole-5-carbonyl)-1,4-dihydropyridine-3-carboxamide and (4R)-4-fluoro-N-[(2R,3S)-4-hydrazinyl-3-hydroxy-1-phenylbutan-2-yl]-2,6-dimethyl-5-(1,2-oxazole-3-carbonyl)-1-(prop-2-en-1-yl)-1,4-dihydropyridine-3-carboxamide (further referred to as EDC and FDC, resp.) were found possessing dual target inhibitory capability. 2D structures of these compounds along with the common moiety are shown in Figure 3(b). The docking results revealed that EDC had the highest XP score of −15.20 kcal/mol against BACE-1 and a significant XP score of −11.92 kcal/mol against AChE. On the other hand, FDC was found to interact with strong binding affinity of −14.39 kcal/mol with BACE-1 and of −11.85 kcal/mol with AChE. Rest of all the docking parameters for these two ligand molecules with respect to both the targets were also taken into consideration and are summarized in Table 3. The pIC_50_ value of both these lead compounds was 6.10 as predicted by the generated GQSAR model. The drug-like properties of the chosen compounds were also taken into account and both of the leads were found to have satisfactory values for all the essential drug-like properties such as logP value and molecular weight which are listed in Table 4.

**Figure 4:**
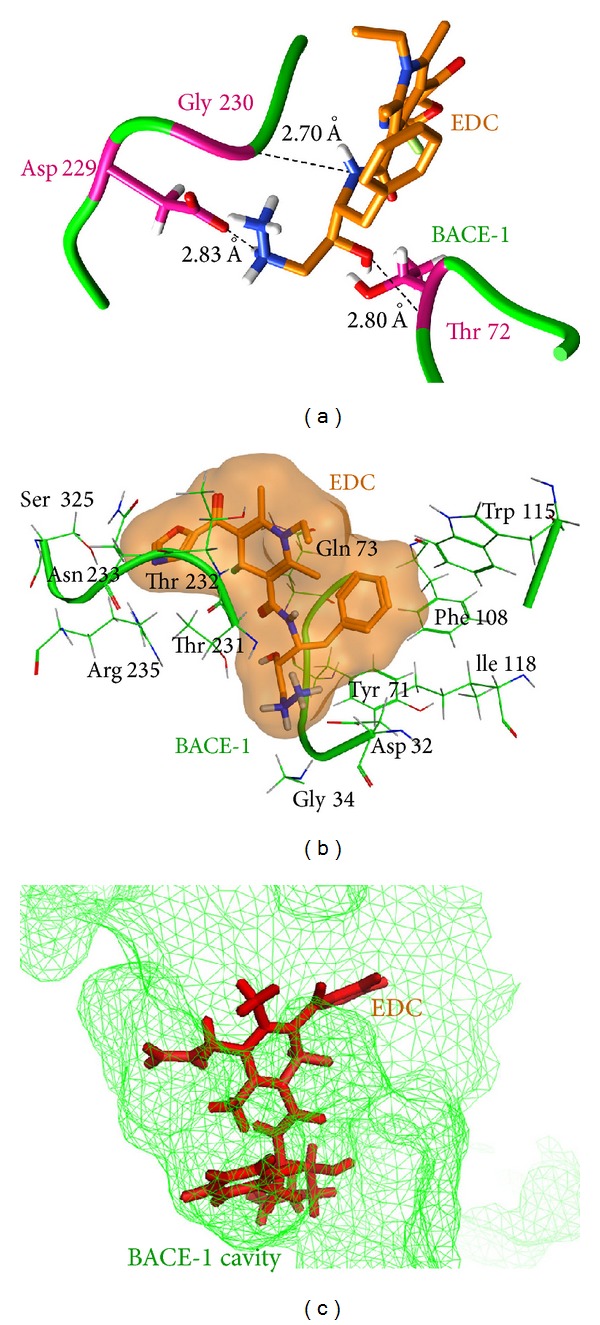
(a) Residues involved in hydrogen bond formation in EDC-BACE-1 complex. (b) Hydrophobically interacting amino acids in EDC-BACE-1 complex. (c) EDC bound in the active site of BACE-1.

*EDC-BACE-1 Complex*. In case of EDC-BACE-1 complex, EDC was found interacting with active site residues (Asp32, Gln73, Asp228, Gly230, Thr232, Asn233, and Arg235) of BACE-1 [4] with formation of four hydrogen bonds and 12 hydrophobic contacts. Among the residues lining the binding site, Asp228 and Gly230 were found participating in hydrogen bond formation with the ligand. The other residue participating in H bond formation was Thr72. The residues Asp32, Gln73, Thr232, Asn233, and Arg235 of the binding cleft along with numerous neighbouring amino acids, namely, Gly34, Tyr71, Phe108, Trp115, Ile118, Thr231, and Ser325, were observed to be involved in hydrophobic interactions with EDC. The involvement of binding site residues of BACE-1 with EDC would block the BACE-1 APP interaction, thereby preventing the processing of APP for A*β* plaque formation. The binding mode of interactions can be well understood through the pictorial representation as shown in Figure 4.

**Figure 5:**
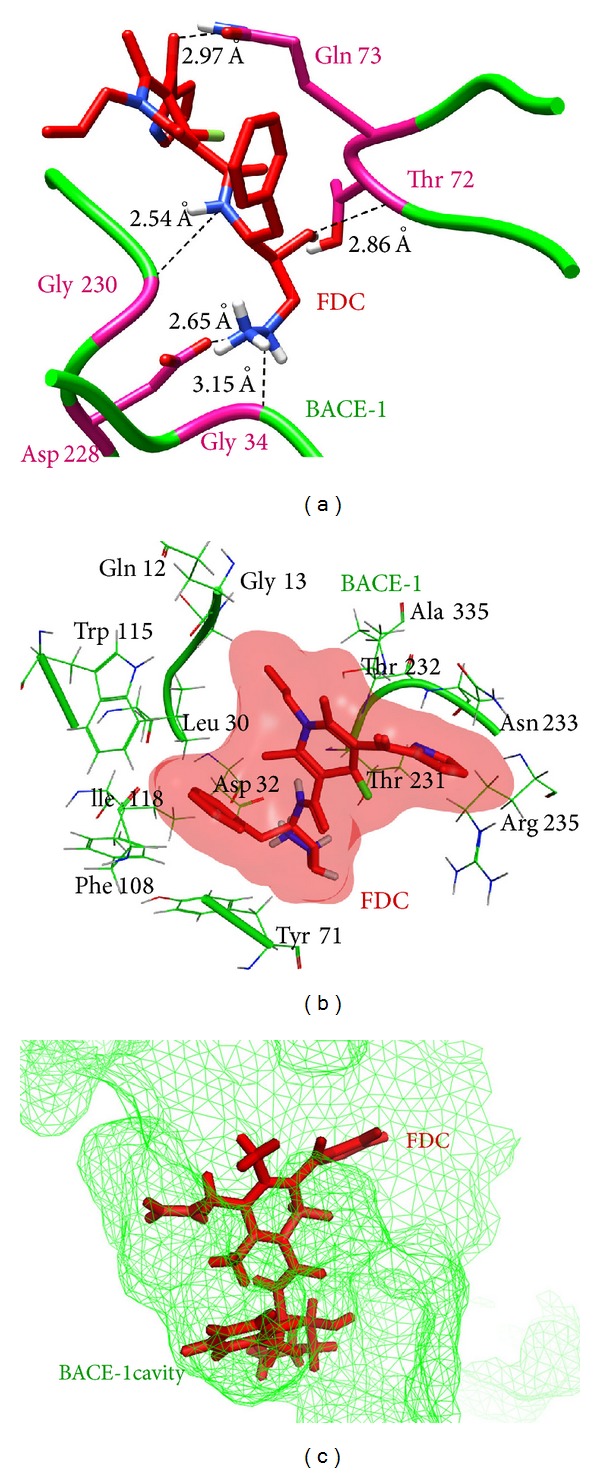
(a) Hydrogen bonds involved in the binding of FDC with BACE-1. (b) Residues of BACE-1 involved in the formation of hydrophobic contacts with FDC. (c) Binding of FDC molecule inside the cavity of BACE-1.

*FDC-BACE-1 Complex*. Interaction analysis of this complex showed 5 hydrogen bonds and 13 hydrophobic interactions between FDC and the binding site residues of BACE-1 as well as with some neighbouring amino acids that can be seen in Figure 5. BACE-1 residues involved in H-bond formation included Gly34, Thr72, Gln73, Asp228, and Gly230. Amino acids, Gln12, Gly13, Leu30, Asp32, Tyr71, Trp115, Ile118, Phe108, Thr231, Thr232, Asn233, Arg235, and Ser325, were making hydrophobic contacts. Binding of the ligand at this site would lead to blocking of protein-protein interactions between BACE-1 and APP.

**Figure 6:**
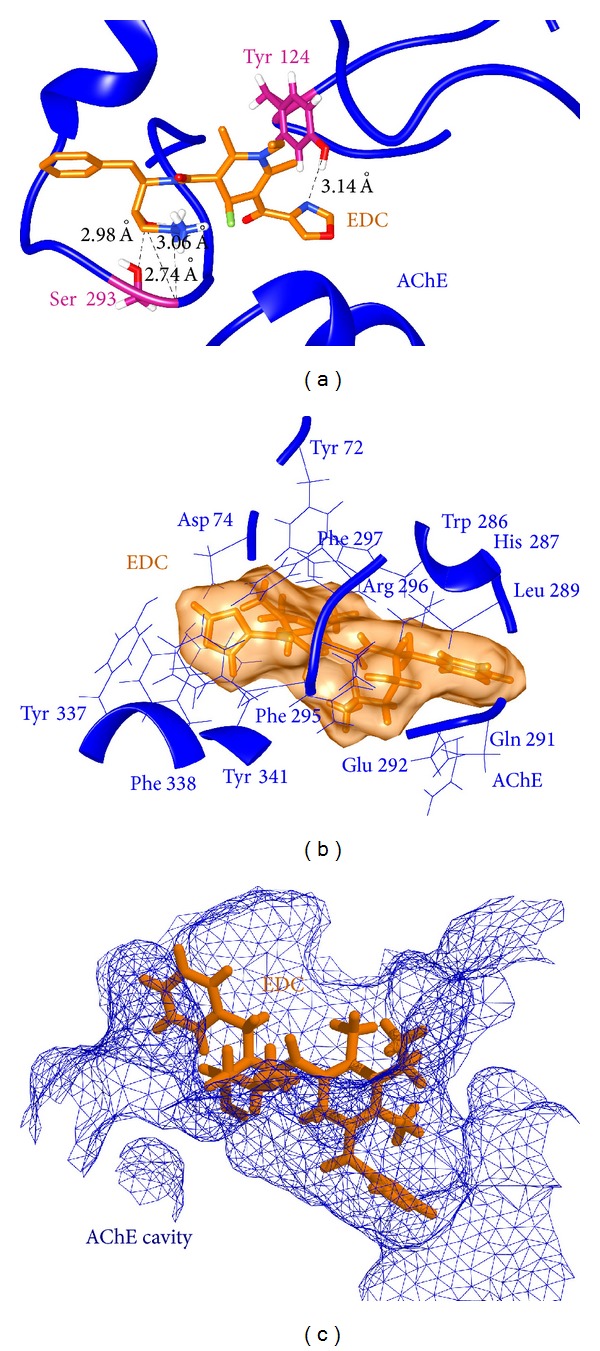
(a) Hydrogen bonds and their lengths as found in EDC-AChE complex. (b) Hydrophobic contacts formed between AChE residues and EDC ligand. (c) Binding of EDC molecule inside the peripheral anionic gorge of AChE.

*EDC-AChE Complex*. Since EDC was evaluated as a dual inhibitor of two different targets BACE-1 and AChE, the mechanistic mode of interaction was also analysed for EDC-AChE complex. In this complex, EDC was observed to form four hydrogen bonds and numerous hydrophobic contacts with PAS residues [23] along with some other surrounding amino acids. Two amino acids Tyr124 and Ser293 were involved in the formation of hydrogen bonds. The residues involved in hydrophobic contacts were Tyr72, Asp74, Trp286, His287, Leu289, Gln291, Glu292, Phe295, Arg296, Phe297, Tyr 337, Phe338, and Tyr341. Convincing docking score and high number of hydrogen bonds as well as hydrophobic interactions suggested EDC to be a significant inhibitor of AChE. Binding of EDC within the PAS of AChE is illustrated in Figure 6.

**Figure 7:**
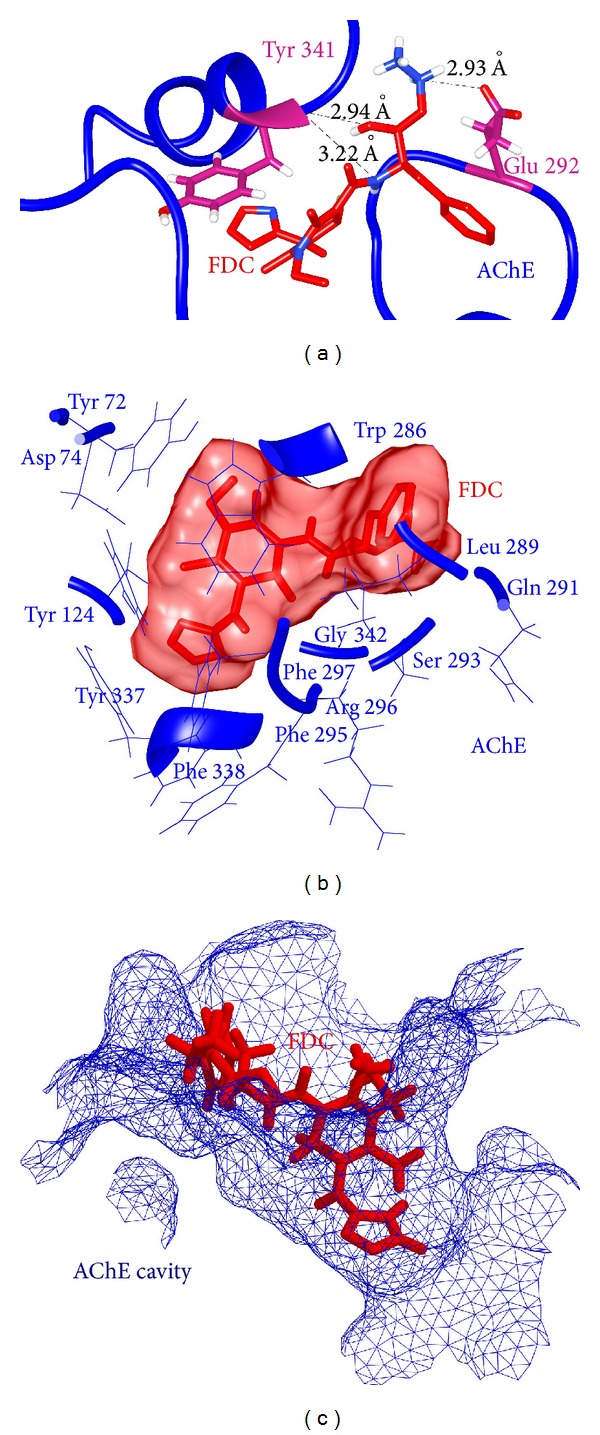
(a) Observed hydrogen bonds with their respective bond length in FDC-AChE complex. (b) FDC surrounded by hydrophobically interacting residues of AChE. (c) Ligand binding inside the PAS of FDC-AChE complex.

*FDC-AChE Complex*. Similar to EDC, the second lead molecule FDC was also evaluated for its dual inhibition property. Docking analysis for FDC-AChE complex showed that FDC was interacting with the PAS cavity of AChE. For this docked complex, three hydrogen bonds formed by two AChE residues (Glu292 and Tyr341) and FDC atoms were detected. A total of 13 hydrophobic contacts were identified with residues Tyr72, Asp74, Tyr124, Trp286, Leu289, Gln291, Ser293, Phe295, Arg296, Phe297, Tyr337, Phe338, and Gly342. The interaction mode of FDC-AChE complex showing hydrogen bonds with their respective bond length and hydrophobic interactions is illustrated in Figure 7.

## 4. Conclusion

This study is an attempt to identify novel dual inhibitors targeting BACE-1 and AChE enzymes. Structural characteristics of a set of dihydropyridine derivatives were studied using a novel group-based QSAR analysis. The GQSAR analysis revealed the importance of 2D descriptors and showed that the chemical group variations in the molecules substantially influenced their biological activity. We also generated a large combinatorial library of 86400 compounds by carrying out substitutions at four different sites of DHP. GQSAR model was utilized further for activity prediction of prepared combinatorial library. The two compounds (EDC and FDC) having high predicted inhibitory activity and the highest docking scores against both of the targets were identified as possessing dual inhibitory properties. We have also provided mechanistic insights into the binding mode of action of these leads. The enhanced predicted activity, high binding score, and the presence of crucial drug like molecular properties provide substantial evidence for consideration of these compounds as potent dual inhibitors for future prospective of AD treatment. This information could be of high value for design and development of novel multitargeted drugs against AD possessing improved binding properties and low toxicity to human cells.

## Supplementary Material

Supplementary Table 1: Chemical structure of the molecules used to build and validate the GQSAR model along with their reported pIC_50_ values. There were 15 compounds in the training set and 5 compounds in the test set.
Supplementary Table 2: Actual and predicted pIC_50_ value along with the values of calculated descriptors for each molecule of the dataset.

## Figures and Tables

**Table 1:** Unicolumn statistical parameters for the selected biological dataset.

	Average	Max.	Min.	Std. dev.	Sum
Training set	4.74	5.10	4.50	0.20	71.13
Test set	4.65	4.83	4.41	0.17	23.27

**Table 2:** Statistical parameters of generated GQSAR model.

Statistical parameter	Value
*r* ^2^	0.85
*q* ^2^	0.68
*F*-test	34.39
*r* ^2^ se	0.08
*q* ^2^ se	0.12
pred_*r* ^2^	0.75
pred_*r* ^2^se	0.1
*Z*score*R* ^2^	5.29
Best rand *R* ^2^	0.52

**Table 3:** Docking parameters for the complexes chosen after Lipinski filter.

Complexes	Glide XP score (kcal/mol)	Glide Evdw (kcal/mol)	Glide Ecoul (kcal/mol)	Glide Emodel (kcal/mol)	Glide Energy (kcal/mol)
EDC-BACE	−15.20	−32.97	−23.79	−96.58	−56.76
FDC-BACE	−14.39	−28.88	−30.66	−97.88	−59.55
EDC-AChE	−11.92	−46.03	−18.82	−106.16	−64.85
FDC-AChE	−11.85	−42.17	−16.71	−86.54	−58.88

**Table 4:** Molecular properties of two top scoring compounds.

Molecular properties	Molecules	
	EDC	FDC
log⁡*P*	1.21	1.54
HBD	4	4
HBA	7	7
Mol. wt. (Dalton)	471.52	483.53
Mol. refractivity	124.69	129.09

HBD: hydrogen bond donar; HBA: hydrogen bond acceptor; Mol.: molecular; wt.: weight.
