# Supplementary material for: Development of Dual Inhibitors against Alzheimer's Disease Using Fragment-Based QSAR and Molecular Docking
Source: Biomed Res Int. 2014 Jun 12;2014:979606. doi: 10.1155/2014/979606 (PMC4075005; doi:10.1155/2014/979606)
Supplement: Supplementary file 1 — Supplementary Table 1: Chemical structure of the molecules used to build and validate the GQSAR model along with their reported pIC50 values. There were 15 compounds in the training set and 5 compounds in the test set. Supplementary Table 2: Actual and predicted pIC50 value along with the values of calculated descriptors for each molecule of the dataset. [file 979606.f1.pdf]

Supplementary Tables

Supplementary Table 1

S. No.	Molecular Structure (2D)	pIC_{50} (nm)
**Training Set**		
1	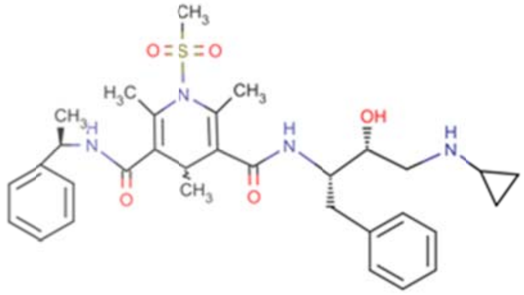	4.80
2	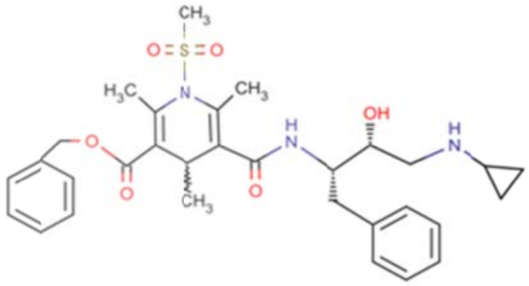	5.02
3	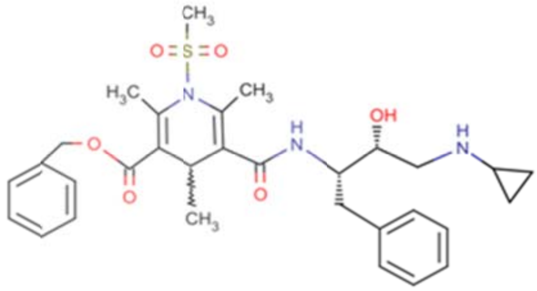	5.10
4	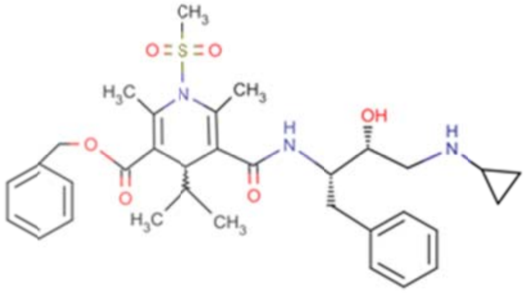	4.90

5	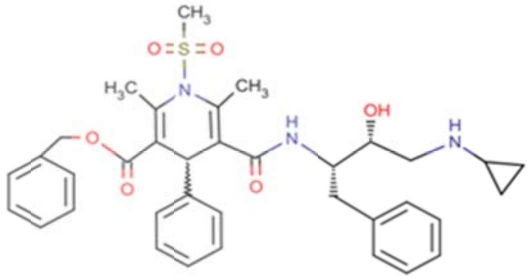	4.70
6	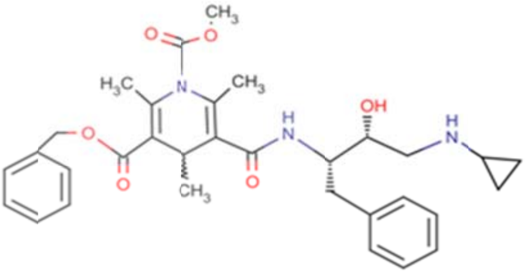	4.82
7	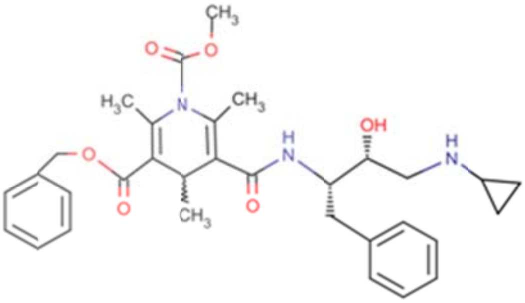	4.81
8	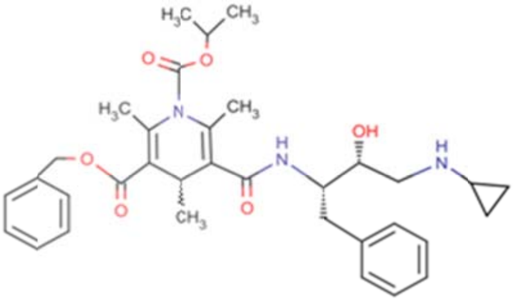	5.05
9	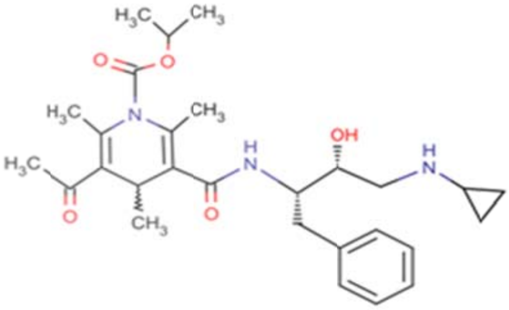	4.53

10	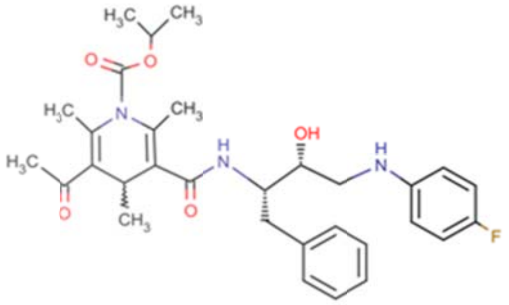	4.60
11	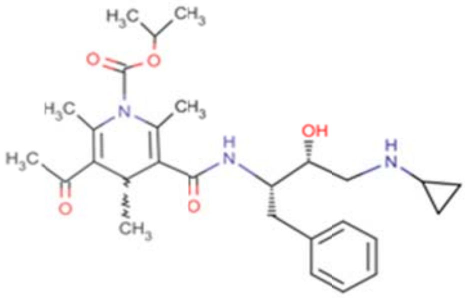	4.50
12	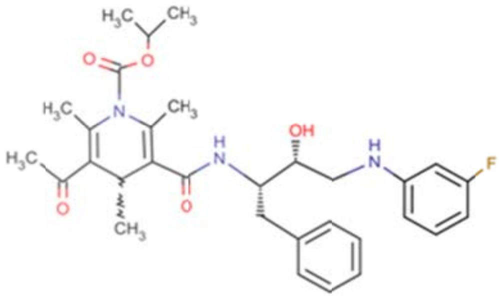	4.60
13	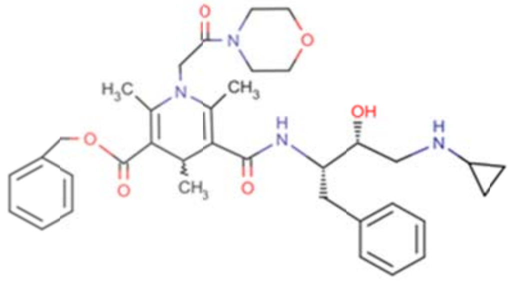	4.55
14	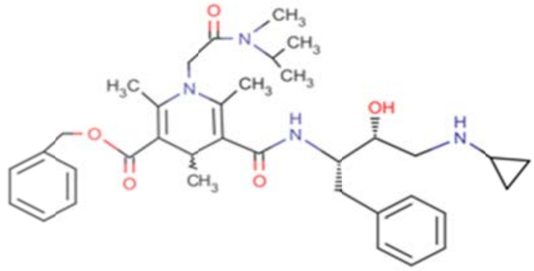	4.55

15	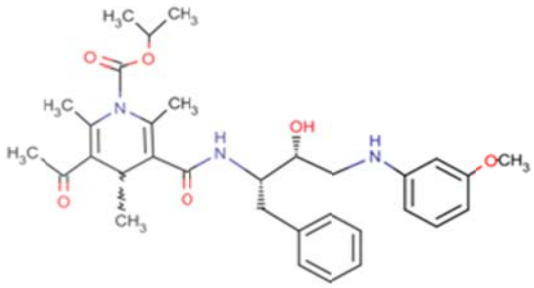	4.60
**Test Set**		
1	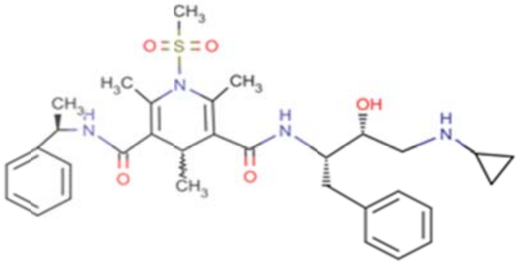	4.80
2	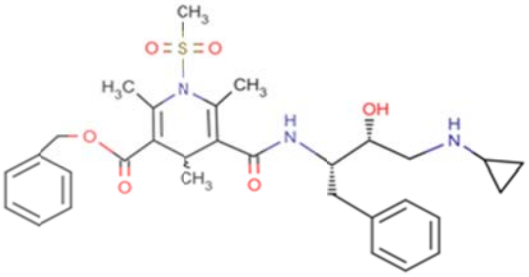	4.83
3	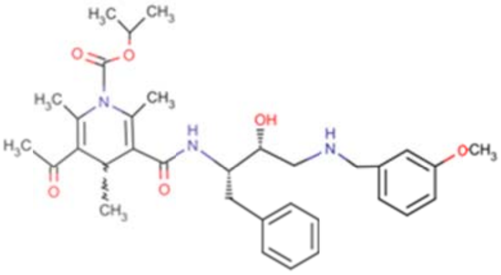	4.63
4	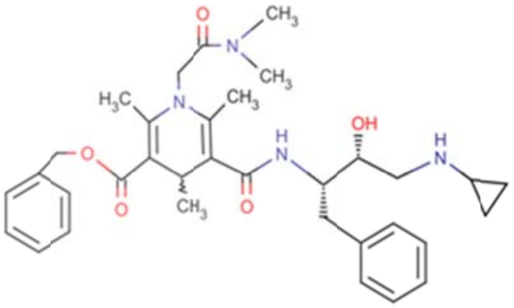	4.60

5	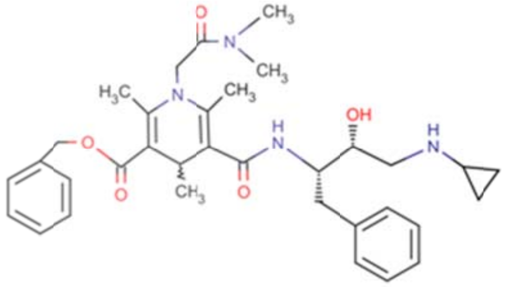	4.41

Supplementary Table 2

S. No.	Actual activity (pIC_{50} in nm)	Selected Descriptors			Predicted activity (pIC_{50} in nm)
		R1-NitrogenCount	R2-DeltaEpsilonA	R3-k3alpha	
1	4.80	1	0	0	4.80
2	4.80	1	0	0	4.80
3	4.83	0	0	0	4.95
4	5.02	0	0	0	4.95
5	5.10	0	0	0	4.95
6	4.90	0	0	0	4.95
7	4.70	0	0.067	0	4.71
8	4.82	0	0	3.63	4.95
9	4.81	0	0	5.01	4.95
10	5.05	0	0	5.63	4.95
11	4.53	0	0	5.63	4.57
12	4.60	0	0	5.63	4.57
13	4.50	0	0	5.63	4.57
14	4.60	0	0	5.63	4.60
15	4.60	0	0	5.63	4.57
16	4.63	0	0	5.63	4.57
17	4.60	0	0	2.63	4.54
18	4.41	0	0	2.63	4.54
19	4.55	0	0	1.77	4.54
20	4.55	0	0	2.52	4.54
